# Report from the CVOT Summit 2020: new cardiovascular and renal outcomes

**DOI:** 10.1186/s12933-021-01254-1

**Published:** 2021-03-31

**Authors:** Oliver Schnell, Xavier Cos, Francesco Cosentino, Thomas Forst, Francesco Giorgino, Hiddo J. L. Heersprink, Mikhail Kosiborod, Christoph Wanner, Eberhard Standl

**Affiliations:** 1 Forschergruppe Diabetes e. V., Ingolstaedter Landstraße 1, 85764 Neuherberg (Munich), Germany; 2 Sant Marti de Provençals Primary Care Centres, Barcelona, Spain; 3 Karolinska University Hospital, Solna, Stockholm, Sweden; 4 CRS Clinical Research Services Mannheim GmbH, Mannheim, Germany; 5 Department of Emergency and Organ Transplantation, University of Bari Aldo Moro, Bari, Italy; 6 Department of Clinical Pharmacy and Pharmacology, University of Groningen, University Medical Center Groningen, Groningen, Netherlands; 7 Cardiometabolic Center of Excellence, University of Missouri-Kansas City, Kansas, MO USA; 8 Universitätsklinikum Würzburg, Würzburg, Germany

**Keywords:** Diabetes, Cardiovascular disease, Heart failure, Chronic kidney disease, Obesity, VERTIS-CV, EMPEROR-Reduced, DAPA-CKD, FIDELIO-DKD, SGLT2i inhibitor, GLP-1 receptor agonist, Mineralocorticoid receptor antagonist

## Abstract

The 6th Cardiovascular Outcome Trial (CVOT) Summit “Cardiovascular and Renal Outcomes 2020” was the first to be held virtually on October 29–30, 2020. As in previous years, this summit served as reference meeting for in-depth discussions on the topic of recently completed and presented major outcome trials. This year, focus was placed on the outcomes of VERTIS-CV, EMPEROR-Reduced, DAPA-CKD, and FIDELIO-DKD. Trial implications for diabetes management and the impact on new treatment algorithms were highlighted for diabetologists, cardiologists, endocrinologists, nephrologists, and general practitioners. Discussion evolved from major outcome trials using SGLT-2 inhibitors for treatment and prevention of heart failure and chronic kidney disease in people with and without diabetes, to additional therapy options for chronic kidney disease with a novel mineralocorticoid receptor antagonist. Furthermore, challenges in diabetes management like COVID-19 and obesity, as well as novel treatment strategies and guidelines, were discussed.

The 7th Cardiovascular Outcome Trial Summit will be held virtually on November, 18–19, 2021 (http://www.cvot.org).

## Background

Diabetes mellitus is one of the fastest growing global health emergencies of the twenty-first century and has reached alarming levels. In the last 20 years, the estimated prevalence of diabetes (type 1 and type 2 combined) has risen from 151 million (4.6% of the global population) in the year 2000, to 463 million (9.3%) today [1]. By 2045, the International Diabetes Federation (IDF) estimates an increase in the number of people with diabetes to 700 million (10.9%), with moderate increase in Europe (15%) and North America (33%) and high increase in South East Asia (74%), the Middle East (96%), and Africa (143%) [2]. Furthermore, diabetes affects especially low and middle income countries, as 77% of all people with diabetes worldwide live in those countries [3]. About 90% of the adults with type 2 diabetes mellitus (T2D) have at least one comorbid condition, each with their own risks and challenges. A recent systematic literature analysis, including over 4.5 million people with T2D, revealed that approximately 32% were affected by cardiovascular diseases (CVD). In detail, the study showed a prevalence of ≈29% for atherosclerosis, ≈21% for coronary heart disease, ≈15% for heart failure (HF), ≈10% for myocardial infarction (MI), and ≈7.5% for stroke [4]. Similarly, at least 40% of persons with T2D developed diabetic kidney disease (DKD) as leading cause of chronic kidney disease (CKD) and end-stage kidney disease (ESKD) [5]. CVD, CKD, and diabetes represent leading global causes of death, showing more than 25% increase for CVD associated deaths and nearly twofold increase for CKD and diabetes associated deaths since 1990. T2D results in a reduced life expectancy by 10 years with CVD and by 16 years with CKD, the latter being the most prominent comorbidity in T2D [6].

Rising concerns of potentially higher risk for cardiovascular (CV) events associated with some glucose-lowering medications was one of the contributing factors for the guidance from the US Food and Drug Administration (FDA) on the assessment of the cardiovascular safety of newer glucose-lowering drugs in 2008 [7]. As the result, a number of novel agents were evaluated in long-term cardiovascular outcome trials (CVOTs). Several major outcome trials for three glucose-lowering classes have been conducted for people with T2D: glucagon-like peptide-1 (GLP-1) receptor agonists, dipeptidyl peptidase-4 (DPP-4) inhibitors, and sodium-glucose co-transporter-2 (SGLT2) inhibitors. These major outcome trials included 17 CVOTs up to and including 2019: seven trials of GLP-1 receptor agonists [8–14], five trials of DPP-4 inhibitors [15–19], four trials of SGLT2 inhibitors [20–24] with an additional SGLT2 inhibitor trial for HF [25]. Furthermore, some of these trials also published data on kidney outcomes, although these were secondary endpoints or exploratory analyses. An exception was the CREDENCE trial published in 2019, which was designed with kidney outcomes as its primary endpoint [26].

Kidney function is typically evaluated by estimation of glomerular filtration rate (eGFR) based on serum creatinine measurement. Kidney damage is analysed by urine albumin-to-creatinine ratio (UACR) determination in a morning sample [27].

Outcome trials with GLP-1 receptor agonists demonstrated beneficial effects on albuminuria, while SGLT2 inhibitors showed a reduction in both albuminuria and “hard” kidney outcomes. As a trial primarily powered for kidney outcomes in patients with T2D and established DKD, CREDENCE showed positive effects for the SGLT2 inhibitor canagliflozin on such outcomes [26].

In 2020, the list of SGLT2 inhibitor major outcome trials in diabetes was expanded by two CVOTs (VERTIS-CV [23]—Ertugliflozin and SCORED [28]—Sotagliflozin), one renal outcome trial (DAPA-CKD [29]—Dapagliflozin), and two heart failure trials (EMPEROR-Reduced [30]– Empagliflozin and SOLOIST-WHF [31]—Sotagliflozin). In addition, a trial of a novel mineralocorticoid receptor antagonist (MRA) for renal outcomes (FIDELIO-DKD [32]—Finerenone) was published.

As in previous years [33–37], we present and summarise key aspects discussed at the sixth edition of the CVOT Summit in October 2020, which was the first to be held virtually. The CVOT Summit—Cardiovascular and Renal Outcomes 2020 was an interdisciplinary platform, which was also organized in conjunction with four study groups: Primary Care Diabetes Europe (PCDE, www.pcdeurope.org), European Diabetic Nephropathy Study Group (EDNSG, www.ednsg.org), the Incretin Study Group (www.easd-incretin.ku.dk), and the Working Group Diabetes & Herz (www.ddg.org). Participants from five continents with specialities in diabetology, endocrinology, cardiology, nephrology, and primary care contributed to the discussions of the Virtual CVOT Summit on Cardiovascular and Renal Outcomes 2020 (www.cvot.org).

### Updates on CVOTs

A summary of characteristics and results of kidney, HF and CV outcome trials published in 2020 is listed in Tables 1, 2, 3, 4.

**Table 1:** Overview of basic characteristics of kidney, heart failure and cardiovascular outcome studies completed in 2020

Study name	Study status	Drug	Drug class	Intervention	Primary outcome	n	Follow up [years]	Start and end date	Clinicaltrials.gov ID
VERTIS-CV [23]	Completed	Ertugliflozin	SGLT-2 inhibitor	Ertugliflozine 5 mg or 15 mg once daily vs. placebo	Composite outcome of MACE (CV death, nonfatal MI, nonfatal stroke)	8246	3.5	11.2013–12.2019	NCT01986881
DAPA-CKD [29]	Completed	Dapagliflozin	SGLT-2 inhibitor	Dapagliflozine 10 mg once daily vs. placebo	Composite of sustained ≥ 50% eGFR decline, end-stage kidney disease, and renal or CV death	4304	2.4	01.2017–08.2020	NCT03036150
EMPEROR-Reduced [30]	Completed	Empagliflozin	SGLT-2 inhibitor	Empagliflozine 10 mg once daily vs. placebo	Composite of CV death or hospitalisation for HF	3730	1.3	02.2017–08.2020	NCT03057977
FIDELIO-DKD [32]	Completed	Finerenone	Mineralocorticoid receptor antagonist	Finerenone 10 mg or 20 mg once daily vs. placebo	Composite of onset of kidney failure, sustained ≥ 40% eGFR decline or renal death	5734	2.6	07.2015–04.2020	NCT02540993
SOLOIST-WHF [31]	Completed	Sotagliflozin	SGLT-2 Inhibitor	Sotagliflozin 200 mg or 400 mg once daily vs. placebo	Total occurrences of CV death, HHF, and urgent visits for HF	1222	0.7	06.2018–06.2020	NCT03521934
SCORED [28]	Completed	Sotagliflozin	SGLT-2 Inhibitor	Sotagliflozin 200 mg or 400 mg once daily vs. placebo	Total occurrences of CV death, HHF, and urgent visits for HF	10584	1.3	11.2017–07.2020	NCT03315143

**Table 2:** Cardiovascular outcome trials completed in 2020: comparison of active vs. placebo group

VERTIS-CV [23]		SCORED [28]	
Class & cardiovascular outcomes	HR (95.6% CI) p-value	Class & cardiovascular outcomes	HR (95.6% CI) p-value
*Primary composite outcome* Composite outcome of MACE (CV death, nonfatal MI, nonfatal stroke)	0.97 (0.85–1.11) p < 0.001	*Primary composite outcome* Total occurrences of CV death, HHF, and urgent visits for HF	0.74 (0.63–0.88) p < 0.001
*Secondary outcome* CV death or HHF	0.88 (0.75–1.03) p = 0.11	*Secondary outcome* Total occurrence of HF events	0.67 (0.55–0.82) p < 0.001
*Secondary outcome* CV death	0.92 (0.77–1.11) p = 0.39	*Secondary outcome* CV death	0.90 (0.73–1.12) p = 0.35
*Secondary outcome* Hospitalization for heart failure	0.70 (0.54–0.90) p = 0.006	*Secondary outcome* 3P-Mace and HHF events	0.72 (0.63–0.83)
*Secondary outcome* Kidney composite: dialysis/transplant, doubling of serum creatinine level, or renal death	0.81 (0.63–1.04) p = 0.08	*Secondary outcome* All-cause mortality	0.99 (0.83–1.18)

Adverse events	Event rate (%) active vs. placebo group	Adverse events	Event rate (%) active vs. placebo group
Urinary tract infection	12.2 vs. 12.0 vs. 10.2	Diarrhea	8.5 vs. 6.0
Acute pancreatitis	0.4 vs. 0.2 vs. 0.4	Genital infections	2.4 vs. 0.9
Diabetic ketoacidosis	0.3 vs. 0.4 vs. 0.1	Diabetic ketoacidosis	0.6 vs. 0.3

**Table 3:** Kidney outcome trials completed in 2020: comparison of active vs. placebo group

DAPA-CKD [29]		FIDELIO-DKD [32]	
Class & cardiovascular/Kidney outcomes	HR (95.6% CI) p-value	Class & cardiovascular/Kidney outcomes	HR (95.6% CI) p-value
*Primary composite outcome* Composite of sustained ≥ 50% eGFR decline, end-stage kidney disease, and renal or CV death	0.61 (0.51–0.72) p = 0.0000000028	*Primary composite outcome* Composite of onset of kidney failure, sustained ≥ 40% eGFR decline or renal death	0.82 (0.73–0.93) p = 0.001
*Secondary outcome* Sustained ≥ 50% eGFR decline, ESKD or renal death	0.56 (0.45–0.68) p = 0.0000000018	*Secondary outcome* Composite of CV death, nonfatal MI, nonfatal stroke, and HHF	0.86 (0.75–0.99) p = 0.03
*Secondary outcome* Chronic dialysis, kidney transplantation, renal death	0.66 (0.49–0.90) p = 0.0072	*Secondary outcome* All-cause mortality	0.90 (0.75–1.07)
*Secondary outcome* CV death or HHF	0.71 (0.55–0.92) p = 0.0089	*Secondary outcome* Hospitalisation for any cause	0.95 (0.88–1.02)
*Secondary outcome* All-cause mortality	0.69 (0.53–0.88) p = 0.0035		

Adverse events	Event rate (%) active vs. placebo group	Adverse events	Event rate (%) active vs. placebo group
Kidney event	7.2 vs. 8.7	Hypertension	7.5 vs. 9.6
Diabetic ketoacidosis	0.0 vs. 0.1	Hyperkalaemia	15.8 vs. 7.8
Volume depletion	5.9 vs. 4.2	Hypertension	7.5 vs. 9.6

**Table 4:** Heart failure outcome trials completed in 2020: comparison of active vs. placebo group

EMPEROR-reduced [30]		SOLOIST-WHF [31]	
Class & Cardiovascular outcomes	HR (95.6% CI) p-value	Class & Cardiovascular outcomes	HR (95.6% CI) p-value
*Primary composite outcome* Composite of CV death or hospitalisation for HF	0.75 (0.65–0.86) p < 0.001	*Primary composite outcome* Total occurrences of CV death, HHF, and urgent visits for HF	0.67 (0.52–0.85) p < 0.001
*Secondary outcome* Total no. of hospitalisations for HF	0.70 (0.58–0.85) p < 0.001	*Secondary outcome* Total occurrence of HF events	0.64 (0.49–0.83) p < 0.001
*Secondary outcome* Mean slope of change in eGFR — ml/min/1.73 m^2^ per year	1.73 (1.10–2.37) p < 0.001	*Secondary outcome* CV death	0.84 (0.58–1.22) p = 0.36
*Other prespecified analyses* Composite kidney outcome	0.50 (0.32–0.77)	*Secondary outcome* 3P-Mace and HHF events	0.72 (0.56–0.92)
*Other prespecified analyses* No. of hospitalisation for any cause	0.85 (0.75–0.95)	*Secondary outcome* All-cause mortality	0.82 (0.59–1.14)
*Other prespecified analyses* All-cause death	0.92 (0.77–1.10)		

Adverse events	Event rate (%) active vs. placebo group	Adverse events	Event rate (%) active vs. placebo group
Worsening renal function	3.2 vs. 5.1	Diarrhea	6.1 vs. 3.4
Genital tract infections	1.7 vs. 0.6	Severe hypoglycaemia	1.5 vs. 0.3
Hypotension	9.4 vs. 8.7	Hypotension	6.0 vs. 4.6

#### SGLT2 inhibitors

#### VERTIS-CV (Table 2: cardiovascular outcomes)

The VERTIS-CV trial [23] investigated effects of ertugliflozin (5 mg or 15 mg/daily) in 8246 patients (≥ 40 years old) with T2D and established atherosclerotic cardiovascular disease (ASCVD) with a mean follow-up time of 3.5 years. The primary endpoint (non-inferiority) was a composite outcome of major adverse cardiovascular events (MACE) comprising CV death, nonfatal MI, and nonfatal stroke (3P-MACE). The key secondary endpoints (superiority) were (a.) composite outcome of CV death/ hospitalization for heart failure (HHF), (b.) CV death, and (c.) kidney composite outcome (renal death, ESKD—dialysis/transplant, doubling of serum creatinine) [23].

Ertugliflozin achieved its primary endpoint, affirming non-inferiority for MACE (HR 0.97 [95.6% CI 0.85–1.11]; p < 0.001) over placebo. The key secondary composite endpoint of CV death or HHF did not differ significantly between groups (HR 0.88 [95.8% CI 0.75–1.03]; p = 0.11 for superiority), nor did CV death (HR 0.92 [95.8% CI 0.77–1.11]; p = 0.39), but a 30% lower risk of HHF was observed with ertugliflozin (HR 0.70 [95.8% CI 0.54–0.90]; p = 0.006). The kidney composite outcome was 19% lower, yet not statistically significant (HR 0.81 [95.8% CI 0.63–1.04]; p = 0.08) [23]. A pre-specified analysis showed that the subgroups of patients with the greatest reduction of HF-related events were those with an estimated glomerular filtration rate (eGFR) below 60 mL/min/1.73 m^2^ and those with micro- and macro-albuminuria [38].

In terms of adverse events, ertugliflozin was generally safe and well tolerated with known risks for the SGLT2 inhibitor class, including genital mycotic infections. Acute kidney injury, diabetic ketoacidosis (DKA) and amputation were balanced between the groups[23].

#### DAPA-CKD (Table 3: kidney outcomes)

The DAPA-CKD trial [29] assessed whether treatment with dapagliflozin (10 mg/daily) reduces the risk of kidney and CV events in 4304 people with CKD, with or without T2D. Patients with CKD who had an eGFR ≥ 25 to ≤ 75 mL/min/1.73m^2^ and a UACR ≥ 200 to ≤ 5000 mg/g (22.6 to 564 mg/mmol) were enrolled. In addition, all patients were maintained on a stable and individualized maximum tolerated dose of an angiotensin-converting-enzyme (ACE) inhibitor or angiotensin-receptor blocker (ARB) for at least 4 weeks. Two-third of participants had a diagnosis of T2D. The primary outcome was a composite of sustained ≥ 50% eGFR decline, ESKD, and renal or CV death. Secondary outcomes (in hierarchical order) encompassed (a.) a composite outcome of sustained ≥ 50% eGFR decline, ESKD or renal death, (b.) CV death or HHF, and (c.) all-cause mortality. The trial was stopped early for efficacy based on a recommendation from the independent Data Monitoring Committee following a regular review meeting [29].

During a median of 2.4 years follow-up, dapagliflozin significantly reduced the primary composite outcome by 39% (HR 0.61 [95% CI 0.51–0.72]; p < 0.0001), with risk reduction for ESKD (HR 0.64 [95% CI 0.50–0.82]; p < 0.0004), CV death (HR 0.81 [95% CI 0.58–1.12]; p < 0.203), and a sustained eGFR decline ≥ 50% (HR 0.53 [95% CI 0.42–0.67]; p < 0.0001) compared to placebo. Pre-specified subgroup analysis did not show differences for people with T2D (HR 0.64 [95% CI 0.52–0.79]) versus people without T2D (HR 0.50 [95% CI 0.35–0.72]) (p = 0.24 for interaction). Significant improvement of secondary outcomes was also observed: the risk for CV death or HHF (HR 0.71 [95% CI 0.55–0.92]; p = 0.0089) and all-cause mortality (HR 0.69 [95% CI 0.53–0.88]; p = 0.0035) were all significantly reduced by dapagliflozin [29].

No significant increase of adverse events of interest was observed in the dapagliflozin group compared with the placebo group. Notably, no diabetic ketoacidosis events were reported in patients assigned to dapagliflozin, and patients without diabetes did not experience any severe hypoglycaemic episodes [29].

#### EMPEROR-Reduced (Table 4: HF outcome)

The EMPEROR-reduced trial [30] assessed the effect of empagliflozin (10 mg/daily) in 3730 patients with chronic symptomatic HF, reduced ejection fraction of 40% or less (HFrEF), and elevated natriuretic peptides, with a median follow-up of 1.33 years. Of the 3730 patients enrolled, 50% had T2D, 34% had prediabetes (HbA_1c_ 5.7–6.4%), and 16% had normoglycemia (HbA_1c_ < 5.7%) [39]. The primary endpoint was a composite of CV death or HHF, followed by the first secondary endpoint of the total (first and recurrent) HHF. The key second secondary endpoint was the slope of decline in eGFR over time [30].

Empagliflozin significantly decreased the risk of the primary composite outcome by 25% (HR 0.75 [95% CI 0.65–0.86]; p < 0.001), driven primarily by lower risk of HHF (HR 0.69 [95% CI 0.59–0.81]), with no significant decrease in CV death (HR 0.92, 95% CI 0.75—1.12). Subgroup analysis showed similar treatment benefits in people with diabetes (HR 0.72 [95% CI 0.60–0.87]) and without diabetes (HR 0.78 [95% CI 0.64–0.97]), respectively, *P*-interaction = 0.57 [39]. Significant improvement was observed for the key secondary outcome of total HHF by 30% (HR 0.70 [95% CI 0.58–0.85]; p < 0.001). In addition, the second key secondary outcome showed a slower decline in eGFR with empagliflozin over the treatment period (by 1.7 ml/min/1.73m^2^ per year [95% CI 1.1–2.4]; p < 0.001) compared to placebo. Furthermore, HbA_1c_ did not change in patients without diabetes, and there was no increased risk of hypoglycaemic episodes or DKA with empagliflozin [30].

#### EMPEROR-Reduced vs. DAPA-HF

A comparable decrease in the risk of the composite endpoint of CV death or worsening HF was observed in the DAPA-HF trial [25] (HR 0.74 [95% CI 0.65–0.85]; p < 0.001) and the EMPEROR-Reduced trial [30] (HR 0.75 [95% CI 0.65–0.86]; p < 0.001). Both DAPA-HF (assessing dapagliflozin) and EMPEROR-Reduced (assessing empagliflozin) were combined in a meta-analysis [40] to assess the effects of SGLT2 inhibitors on CV outcomes in patients with HFrEF, with or without diabetes. EMPEROR-Reduced [30] was designed to study the same target population as DAPA-HF, but included individuals with lower ejection fraction and higher natriuretic peptide levels. The combination of 8374 patients from both trials in a meta-analysis showed a 13% reduction in all-cause mortality (pooled HR 0.87 [95% CI 0.77–0.98]; p = 0.018), 14% reduction in CV death (HR 0.86 [95% CI 0.76–0.98]; p = 0.028) [40], and 38% reduction of the composite kidney endpoint (HR 0.62 [95% CI 0.43–0.90]; p = 0.013). Taken together, the combined results of DAPA-HF and EMPEROR-Reduced enrolled a broader spectrum of severity of HF than either trial alone. When data was combined from both trials, reduced risk of the composite endpoint of CV death or HHF and improved kidney outcomes were observed [40].

#### SOLOIST-WHF (Table 4: HF outcomes) and SCORED (Table 2: cardiovascular outcomes)

Two other SGLT2 inhibitor trials evaluated sotagliflozin, which also provides gastrointestinal SGLT1 inhibition, and were published shortly after the Virtual CVOT Summit 2020 [28, 31]. Both trials ended early due to loss of funding from the sponsor. This led to a reduction in power to test for the original primary endpoints. Due to these constraints, as well as evolution of the field since the trials were initiated, led to new primary composite endpoints being pre-specified in the statistical analysis plans prior to database lock. Furthermore, clinical events comprising the primary and secondary endpoints were reported by the site investigators, and not adjudicated.

The SOLOIST-WHF trial assessed the effect of a daily dose of 200 mg sotagliflozin (with a dose increase to 400 mg, depending on tolerability) in 1222 patients with T2D who were either hospitalised for worsening HF or recently discharged, with a median follow-up time of 9 months. The trial was originally designed with a primary endpoint of the first occurrence of either death from CV causes or HHF. The new primary composite endpoint was the total number of deaths from CV causes, HHF and urgent visits for HF [31].

The primary composite outcome was significantly reduced with sotagliflozin versus placebo by 33% (HR 0.67 [95% CI 0.52–0.85]; p < 0.001), with a relative risk reduction of CV death by 16% (HR 0.84 [95% CI 0.58–1.22]) and all-cause death by 18% (HR 0.82 [95% CI 0.59–1.24]). Of importance, there was similar treatment benefit in patients with both reduced (LVEF < 50%: HR 0.72 [95% CI 0.56–0.94]) and preserved (LVEF >  50%: HR 0.48 [95% CI 0.27–0.86]) ejection fraction. In terms of adverse events, patients with sotagliflozin showed higher prevalence for diarrhoea (6.1% vs. 3.4%) and for severe hypoglycaemia (1.5% vs. 0.3%) compared to placebo. There was no excess of DKA events with sotagliflozin versus placebo, despite the fact that this trial evaluated acutely hospitalized or recently discharged patients [31].

The SCORED trial investigated the effect of 200 mg sotagliflozin once daily (with a dose increase to 400 mg, depending on tolerability) in 10,584 patients with T2D, CKD with or without albuminuria (eGFR 25 to 60 mL/min/1.73m^2^), and risk for CVD with a median follow-up time of 16 months. As already described for the SOLOIST-WHF trial, the originally specified primary endpoints in the SCORED trial (first occurrence of MACE and the first occurrence of death from CV causes or HHF) were changed prior to database lock. The new primary endpoint was the total number of deaths from CV causes, HHF and urgent visits for HF [28].

Sotagliflozin significantly reduced the primary endpoint by 26% (HR 0.74 [95% CI 0.63–0.88]; P < 0.001), driven primarily by HF events, with no independent reduction in CV death (HR 0.90 [95% CI 0.73–1.12]; P = 35). Of importance, sotagliflozin also reduced the risk of the original primary endpoints of time to first MACE by 16% (HR 0.84 [95% CI 0.72–0.99]) and time to first CV death or HHF by 23% (HR 0.77 [95% CI 0.66–0.91]; P < 0.001). In terms of adverse events, diarrhoea, genital mycotic infections, volume depletion, and DKA occurred more often in patients receiving sotagliflozin [28].

### Mineralocorticoid receptor antagonists

#### FIDELIO-DKD: (Table 3: kidney outcomes)

The FIDELIO-DKD trial [32] assessed the kidney and CV efficacy and safety of finerenone compared with placebo in patients with T2D and CKD. Finerenone is a novel, third-generation, potent and selective oral, non-steroidal mineralocorticoid-receptor antagonist (MRA) that is associated with lower rates of hyperkalaemia and other typical MRA-associated side effects as compared with steroidal MRAs. MRAs such as spironolactone demonstrated some kidney protective effects, such as reduced albuminuria, and favourable cardiovascular effects in patients with T2D and HF. In comparison to steroidal MRAs, finerenone shows greater MR selectivity, and its non-steroidal structure allows the binding of the MR with high affinity, which mitigates against adverse events like gynecomastia [41, 42].

The FIDELIO-DKD trial included 5734 patients with T2D and CKD. Patients had to have persistently high albuminuria (UACR ≥ 30 but < 300 mg/g) with an eGFR ≥ 25 to ≤ 60 ml/min/1.73 m^2^, and history of diabetic retinopathy, or severe albuminuria (UACR ≥ 300 but < 5000 mg/g) and an eGFR ≥ 25 to ≤ 75 ml/min/1.73 m^2^. All patients were treated with renin-angiotensin system blockade at the maximum tolerated dose. The mean follow-up time was 2.6 years. The primary endpoint was a composite of time to first occurrence of kidney failure, a sustained decrease of eGFR ≥ 40% from baseline, or death from renal causes. The key secondary composite outcome was 3P-MACE or HHF [32].

Finerenone showed a significantly decreased risk of the primary composite outcome by 18% (HR 0.82 [95% CI 0.73–0.93]; p = 0.001), which was generally consistent across the pre-specified subgroups. Significant reduction in the key secondary composite outcome (i.e., 3P-MACE or HHF) was also observed (HR 0.86 [95% CI 0.75–0.99]; p = 0.03) with finerenone. Hyperkalemia-related adverse events (serum potassium level > 5.6 mmol/l) were twice as frequent with finerenone (18.3%) versus placebo (9.0%). Furthermore, patients who received finerenone had a higher mean serum potassium level than those who received placebo with a maximal difference of 0.23 mmol/l [32].

### Key topics discussed during the 6th CVOT Summit

#### Challenges in diabetes management—novel strategies and guidelines

##### SGLT-2-Inhibitors: novel outcome studies and treatment options for diabetes, kidney disease and heart failure

CV disease, HF, and progression of kidney disease are the leading causes of morbidity and mortality in people with T2D. Several guidelines now recommend the use of SGLT2 inhibitors as first-line treatment in patients with established ASCVD, HF or DKD [43–45]. Effects of SGLT2 inhibitors on CV and kidney outcomes are largely consistent across the trials [20–26, 28–31], suggesting a class effect. The greatest, common benefits across all trials are the reduced risk of HHF (≈25–30%) and kidney disease progression (≈40%) [46]. These benefits are independent of baseline ASCVD, prior HF, and occur across a spectrum of baseline eGFR and albuminuria. Furthermore, comparable benefits of weight loss, reduction of blood pressure, and reduction of HbA1c can be found in the whole class of SGLT2 inhibitors [46]. In contrast to that, except for sotagliflozin, the MACE efficacy was generally modest across the class (≈10%), and only the EMPA-REG outcome trial in T2D [20] and DAPA-HF trial in HF [22] found independently significant reductions in CV death. Furthermore, DAPA-CKD showed a considerable reduction in all-cause mortality among patients with CKD.

Despite the introduction of novel treatments such as SGLT2 inhibition, the residual risk of progression to ESKD remains substantial [47]. T2D accompanied with CKD is associated with tubulointerstitial fibrosis and inflammation, with a strong relationship between inflammatory cytokines (KRIS) in the kidney and the risk of ESKD [48]. Current treatment recommendations only address the management of haemodynamic and metabolic factors [49]. The use of drugs that block inflammation and fibrosis may be useful in reducing the risk of CKD progression, as the progression is driven by the combined effects of metabolic, haemodynamic, inflammatory, and fibrotic factors [50]. The novel non-steroidal MRA finerenone has a distinct mechanism of action, targeting inflammation and fibrosis. Together with SGLT2 inhibitors, finerenone could be potentially positioned as combination therapy for CKD, even though extensive data on this combined treatment approach is lacking at present [32].

##### GLP-1 receptor agonists: an update

To this date, 7 CVOTs with GLP-1RAs [8–14] have been published, and their evidence has been analysed by several meta-analyses [51–53]. The class of GLP-1 RAs differs in its structure and duration of action with inconsistent effects on cardiovascular outcomes. It is unclear whether differences in HbA_1C_, weight, hypoglycaemia or other factors such as potency, duration of action, or human GLP versus exendin based GLP may contribute to the observed heterogeneous results among the CVOTs. The group of Kristensen et al. [51], meta-analysing all 7 CVOTs using GLP-1 RAs [8–14], showed a significant 12% risk reduction for both MACE, as well as CV and all-cause mortality, a significant 9% reduction in rates of HHF, and a 17% significantly reduced risk for kidney outcomes driven by albuminuria. Unlike SGLT2i, benefit emerges after approximately 12 months, suggesting effects on vascular disease progression. These findings put forward a class effect of GLP-1 RAs with beneficial effects on atherosclerotic events, mortality, and kidney outcomes in patients with T2D.

##### COVID-19

The year 2020 was largely dominated by the spread of the new severe acute respiratory syndrome coronavirus 2 (SARS-CoV-2). By the end of October 2020, the associated respiratory disease—coronavirus disease 2019 (COVID-19)—has been diagnosed in more than 45 million individuals, resulting in more than 1.1 million deaths [54]. Infection and progression of COVID-19 is characterised by an initial infection phase, followed by a respiratory distress phase and a severe hyperinflammation state [55]. Besides the primary impact on the lungs, causing interstitial pneumonitis and severe acute respiratory distress syndrome (ARDS), COVID-19 also affects multiple other organs, especially the cardiovascular system and the kidneys [56]. Risk of severe infection and mortality is increased by comorbidities such as diabetes, obesity, CVD, hypertension, HF, chronic pulmonary disease, cancer, ESKD, organ transplantation and neurological diseases [57]. Suboptimal glucose control in patients with diabetes may be associated with higher risk of SARS-CoV-2 infection due to several mechanisms, such as increased expression of ACE2 [58]. Its expression by endothelial cells might empower Sars-CoV-2 to infect blood vessels by binding to the ACE2 receptor, which thus enhances the wide spread of the virus [59]. As a result, systemic inflammation and the so-called cytokine storm may damage organs such as the heart, liver, and kidneys [60, 61].

##### CVOT results translated into Primary Care Diabetes Guidelines and Recommendations

To assist primary care physicians, the Primary Care Diabetes Europe has formulated a position statement summarising the current understanding of the available T2D treatment options in various patient populations, especially patients with CVD [62, 63]. The position statement is intended to be a practical counterpart to national and international management guidelines. The statement contains a series of presentations and discussions to identify the key issues in CVD risk management in patients with T2D. These patients are categorized into different risk groups based on individual factors such as ASCVD, HF, CKD, and obesity. Several pharmacotherapeutic agents such as metformin, SGLT2 inhibitors, GLP-1 RA, and DDP-4i are suggested for the different risk groups [62, 63].

##### Primary prevention of CVD in the light of current diabetes guidelines

The ADA and ESC/EASD guidelines underline that treatment targets should be individualised for each patient, by using a multifactorial treatment algorithm for management of complications such as ASCVD, HF, and CKD in T2D [64, 65]. The ADA recommends five key targets that most adults with diabetes should achieve: 1) glycaemic control, 2) lifestyle modification (diet and exercise for weight management), 3) blood pressure control (ACE-I and ARB), 4) management of dyslipidaemia (statins intensified with ezetimibe), and 5) platelet inhibition (aspirin and other anti-thrombosis agents) [66]. In order to reach the HbA_1c_ targets, a high number of glucose-lowering medications is now available, which makes management of hyperglycaemia more complex. Agents such as GLP-1RA and SGLT2 inhibitors are the most prominent glucose-lowering medications in the treatment algorithms for the reduction of MACE in people with T2D, particularly with known ASCVD. The REWIND trial demonstrated a 13% risk reduction of MACE for the GLP-1RA dulaglutide in a population in which 68.5% (6793 patients) did not have established CVD. [67]. Furthermore, the REWIND trial endorses findings from multiple trials supporting the benefit of GLP-1RA for MACE reduction [68]. Therefore, GLP-1RA should be preferably prescribed for patients with ASCVD or at high-risk for CVD, while SGLT2 inhibitors should be prioritised over GLP-1RA for patients with HF or CKD [44].

##### The “living guideline” approach

**Fig. 1:**
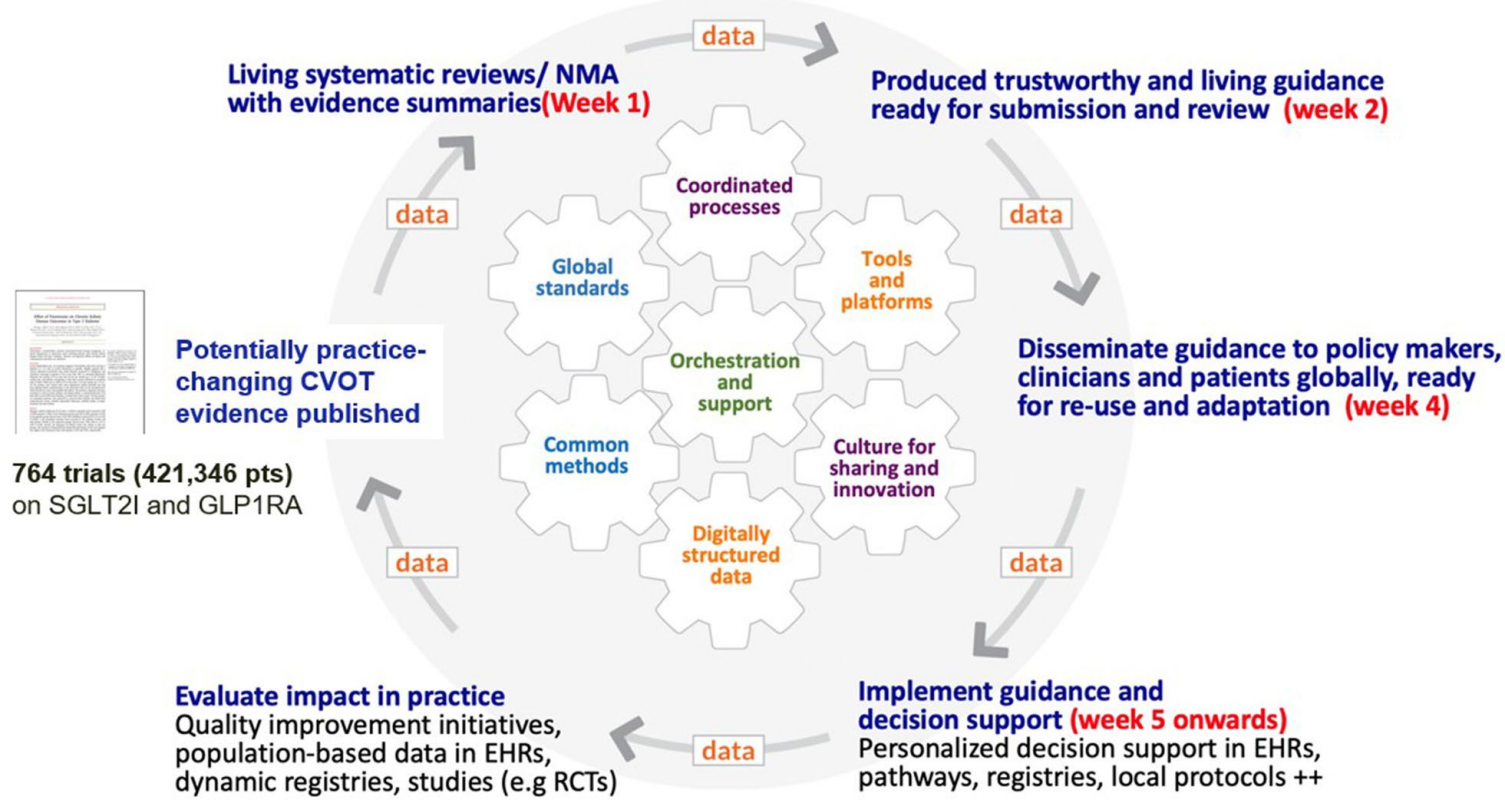
The living guideline model. Living systematic reviews that are constantly updated as new evidence becomes available produce trustworthy guidelines, which are also constantly updated as new evidence that changes direction or strength of recommendations be released (Adapted from: www.magicevidence.org/).

Clinicians and their patients need timely clinical practice guidelines. Guidelines depend on systematic reviews which are difficult to keep up-to-date as research evidence is emerging rapidly. Societies need to apply the best current standards, methods, processes, and platforms for their guidelines to be trustworthy, accessible, and understandable. In 2017, a novel approach termed “Living systematic review” (LSR) was developed. LSRs are reviews that are constantly updated as new evidence becomes available [69]. The advent of LSR enabled the concept of “living guidelines” (Fig. 1), with the possibility to provide timely, up-to-date, and high-quality guidance to clinicians and patients [70].

### Obesity—risk factor and treatment target

Obesity is one of the major health burdens of the twenty-first century reaching pandemic levels. Prevalence of obesity has tripled from 1975 to 2014, with a trend to reaching a global prevalence of 18% in men and surpass 21% in women by the year 2025 [71]. A high body-mass index (BMI) substantially increases the risk of diseases such as T2D, fatty liver disease, hypertension, ASCVD HFpEF, dementia, osteoarthritis, obstructive sleep apnoea, and several cancers [72]. Consequently, obesity contributes to a decline in both the quality of life and life expectancy. During the beginning of the COVID-19 pandemic, obesity as comorbidity appeared to be prominent in about 50% of the hospitalized patients infected with SARS-CoV-2 [73]. In these patients, the risk for hospitalisation, intensive care unit admission, invasive mechanical ventilation requirement, and mortality was increased [74]. The mechanisms causing obesity are not understood well enough to effectively prevent and treat the disease. Treatment is an intensifying lifelong multidisciplinary management with behaviour intervention, pharmacotherapy, and bariatric surgery [75, 76]. Current pharmacological options for weight management in the US and Europe are: (1) the lipase inhibitor orlistat which reduces fat absorption from intestines, (2) the GLP-1RA liraglutide (up to 3.0 mg daily) as appetite suppressor, and (3) the combination of the antidepressant bupropion and the opiate antagonist naltrexone as appetite suppressor [77–79]. The ongoing Semaglutide Treatment Effect in People with obesity (STEP) program evaluates the efficacy and safety of the GLP-1RA semaglutide 2.4 mg subcutaneously once weekly in a broad population. The program consists of 5 trials and provides insights on weight management in people with obesity with and without T2D. Early data from press suggests marked efficacy of semaglutide in reducing body weight [80].

### Cardiometabolic Center of Excellence

Multiple guidelines started in collaboration between international professional groups adopt a selection of glucose-lowering agents based on risk, not just HbA_1c_ control, for T2D treatment [43–45]. Nonetheless, collaborative systems of care are mostly nascent, and the use of therapies with proven outcome benefits remains low [81, 82]. As effective clinical care models have not yet been formed, the Saint Luke’s Haverty Cardiometabolic Center of Excellence has been established in Kansas City to become a new model of care for patients with T2D and CVD, by creating a multi-disciplinary, team based, patient-centered approach to aggressive, comprehensive risk reduction. To maximize CV risk reduction and to reduce morbid events, there is a need for key competencies (standard protocols, patient and staff education) combined with comprehensive treatment plans and research initiatives. This approach may be an opportunity to optimize guidelines and to improve outcomes. The team at Saint Luke’s has also created a national organization, Cardiometabolic Center Alliance (www.cardiometabolicalliance.org) with the purpose of helping other healthcare organizations build their own cardiometabolic centers of excellence, with the ultimate goal of improving quality of care and outcomes in patients with T2D and cardiovascular disease nationwide.

## Conclusion

The sixth edition of the CVOT Summit discussed key results of four recently completed and published major outcome trials in a virtual, interactive, multi-disciplinary format. The summit covered one CVOT (VERTIS-CV), two trials designed to evaluate specifically kidney outcomes (DAPA-CKD and FIDELIO-DKD) and one trial for HF outcomes (EMPEROR-Reduced). In addition, two more CVOTs (SOLOIST-WHF and SCORED) were added to the report, as they were published shortly after the summit was held. The summit provided novel data, insights, strategies, and guidelines for specialists and primary care for cardiovascular and kidney therapy algorithms in people with and without T2D. In-depth discussions and presentations of upcoming kidney and HF trials like FIGARO-DKD, EMPA-KIDNEY, DELIVER, and EMPEROR-Preserved will be resumed at the 7^th^ edition of the CVOT Summit, which will be held virtually on November 18–19, 2021 (https://www.cvot.org).

## Data Availability

Data sharing not applicable to this article as no datasets were generated during the current study.

## Abbreviations

3P(4P)-MACE: 3-Point (4-point) major adverse cardiovascular event
ACE: Angiotensin-converting-enzyme
ARB: Angiotensin-receptor blocker
ASCVD: Atherosclerotic cardiovascular disease
CKD: Chronic kidney disease
CVD: Cardiovascular disease
CV: Cardiovascular
CVOT: Cardiovascular outcome trial
EASD: Study Group
EDNSG: European Diabetic Nephropathy Study Group
DKD: Diabetic kidney disease
DM: Diabetes mellitus
DPP-4i: Dipeptidyl-peptidase 4 inhibitor
EASD: European Association for the Study of Diabetes
ESC: European Society of Cardiology
ESKD: End-stage kidney disease
FDA: US Food and Drug Administration
(e)GFR: (Estimated) glomerular filtration rate
HF: Heart failure
HFpEF: Heart failure with preserved ejection fraction
HFrEF: Heart failure with reserved ejection fraction
HHF: Hospitalisation for heart failure
HR: Hazard ratio
IDF: International diabetes federation
GLP-1 RA: Glucagon-like peptide 1 receptor agonist
LVEF: Left ventricular ejection fraction
MI: Myocardial infarction
MRA: Mineralocorticoid receptor antagonist
PCDE: Primary Care Diabetes Europe
SGLT-2i: Sodium/glucose cotransporter 2 inhibitor
T2D: Type 2 diabetes mellitus
UACR: Urine albumin-to-creatinine ratio
